# Verschluss einer persistierenden urethrorektalen Fistel nach suprasymphysärer Adenomenukleation der Prostata durch kombiniertes Bressel-Naujox- mit einem Gracilisflapverfahren

**DOI:** 10.1007/s00120-022-01924-2

**Published:** 2022-09-06

**Authors:** M. Reichert, P. Schüler, A. Stepniewski, G. Felmerer, L. Trojan, H. C. von Knobloch

**Affiliations:** 1 Klinik für Urologie, Universitätsmedizin Göttingen, Robert-Koch-Str. 40, 37075 Göttingen, Deutschland; 2 Klinik für Allgemein‑, Viszeral- und Kinderchirurgie, Universitätsmedizin Göttingen, Göttingen, Deutschland; 3 Klinik für Unfallchirurgie, Orthopädie und Plastische Chirurgie, Universitätsmedizin Göttingen, Göttingen, Deutschland

**Keywords:** Blasendarmfistel, Fistelchirurgie, Fistelverschluss mit Interponat, Vesikovaginaler Fistelverschluss, Komplikation nach Adenomenukleation der Prostata, Rectovesical fistula, Fistula surgery, Fistula closure with tissue interposition, Vesicovaginal fistula closure, Complications after enucleation of the prostate

## Abstract

Unter Berücksichtigung einiger Grundprinzipien der Fistelchirurgie gibt es verschiedene Möglichkeiten der operativen Sanierung urethrorektaler Fisteln. Ein Standard, welche Operationsmethode unter welchen Umständen eingesetzt werden sollte, gibt es – auch aufgrund der Heterogenität dieser Erkrankung – nicht. Dieser Fall beschreibt die individuelle Adaptation einer Operationstechnik, welche zur Behandlung vesikovaginaler Fisteln eingesetzt wird, auf die Behandlung einer urethrorektalen Fistel eines Patienten nach bereits frustranem Versuch eines Fistelverschlusses, welche auf Basis einer etablierten Methode unter Verwendung eines Interponats zum Erfolg führte.

## Abstract (en)

When taking into consideration the basic principles of fistula surgery, numerous options are available for the surgical repair of rectourethral fistulas. However, there is no standard regarding which surgical method should be used under which circumstances—due to the heterogeneity of this disease. This case report describes the individual adaptation of a surgical technique that is used for the treatment of vesicovaginal fistulas to treat a rectourethral fistula in a patient who had already undergone an unsuccessful fistula closure attempt. Successful closure of the fistula was achieved on the basis of an established method using tissue interposition.

## Anamnese

Ein 69-jähriger Patient stellt sich erstmalig in der UMG in der Klinik für Allgemeinchirurgie aufgrund einer bereits diagnostizierten persistierenden urethrorektalen Fistel (URF) vor. Der Patient leidet u. a. an rezidivierenden Harnwegsinfektionen, Pneumaturie, Fäkalurie, flüssigen Stuhlgang etc.

Zwei Jahre zuvor kam es im Rahmen einer extern durchgeführten suprasymphysären Adenomenukleation nach Freyer und simultaner Blasensteinentfernung zu einer Verletzung der hinteren Prostatakapsel mit Rektumbeteiligung. Bereits im selben Monat erfolgte extern die Relaparotomie mit Übernähung der Prostataloge und transanaler Rektumübernähung bei gestellter Diagnose einer URF. In gleicher Sitzung wurde ein doppelläufiges Sigmoidostoma angelegt. Nach Rückverlagerung des Sigmoidostomas kam es zur Entwicklung einer Peritonitis, was eine erneute Relaparotomie nötig machte. Hierbei wurde ein doppelläufiges Transversostoma angelegt. Zusätzlich entwickelte der Patient subkutane Wundheilungstörungen – einmalig auch mit Faszienbeteiligung.

## Diagnostik

### Urethrozystoskopie (UC).

Es zeigte sich ein Fistelgang aus der prostatischen Harnröhre proximal des externen Schließmuskels. Der Blasenhals-Colliculus-Abstand lag bei ca. 1,5 cm, die Blasenkapazität bei > 500 ml.

### Rektoskopie durch Allgemeinchirurgie.

In der Rektoskopie konnte kein Fistelgang nachgewiesen werden.

### Magnetresonanztomographie (MRT).

Nachweis einer URF mit Kontrastmittelübertritt in das Rektum.

### Retrogrades Urethrozystogramm (RUG).

**Figure 1:**
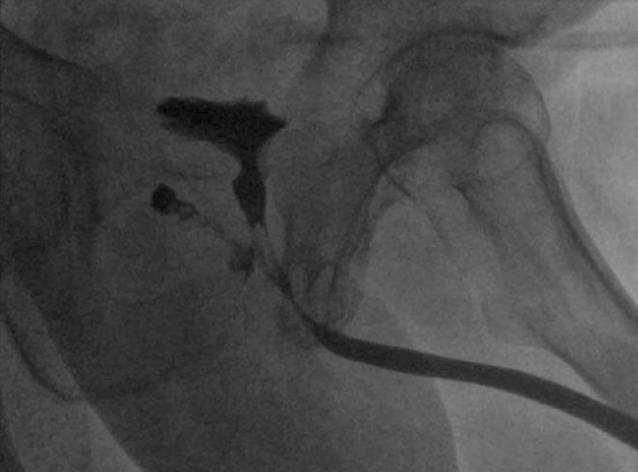

Bestätigung einer URF mit Fistelöffnung unmittelbar proximal des externen Schließmuskels (Abb. 1).

## Therapie und postoperatives Management

### Operation

Zur Sanierung der URF wurde eine mittels Längslaparotomie durchzuführende retropubische Prostatektomie geplant. Es zeigte sich nach Öffnung der Faszie ein stark verwachsener Situs, sowohl intra- als auch extraperitoneal. Ein Versuch der Blasenmobilisierung verlief bei einer Art „frozen pelvis“ frustran. Eine Prostatektomie mit Fistelexzision und -verschluss ohne Zystektomie mit Anlage einer inkontinenten Harnableitung erschien nicht möglich.

Durch eine Sectio alta zeigten sich unauffällige intravesikale Verhältnisse mit orthotopen Ostien beidseits und weitem Blasenhals. Die Prostata war durch ihre tiefe Lage nicht einsehbar. Es erfolgte die protektive Schienung der Harnleiter mit 8‑Ch-Ureterenkatheter (UK) beidseits.

Es wurde die intraoperative Indikation für ein kombiniert abdominales/perineales Vorgehen und Fistelverschluss gestellt, u. a. mit der Verwendung eines Gracilisflaps. Die Wahl des Gracilisflaps als Interponat erschien in diesem Fall nahezu alternativlos, da der intraabdominelle Situs die Verwendung eines Omentumflaps nur unter erhöhtem intraoperativem Risiko zugelassen hätte; sei es durch die Mobilisierung des nahezu nicht mehr vorhandenen Omentums oder durch die von kranial zu erfolgende Präparation des Douglas-Raums.

Ein Peritonealflap wäre aufgrund der Größe der Fistel und deren Komplexität unserer Meinung nach nicht ausreichend gewesen.

Mittels Perinealschnitt wurde eine Schicht zwischen Rektum und Prostata bis zum Douglas-Raum entwickelt und die Fistel exzidiert. Anschließend wurde die Fistelöffnung des Rektums umschnitten und mit Einzelknopfnähten verschlossen.

Zum Verschluss der prostatischen Fistelöffnung wurde ein modifiziertes Verfahren nach Bressel-Naujox gewählt: Die Fistelöffnung zur Prostata wurde umschnitten und eine Tabaksbeutelnaht angelegt. Die Fadenenden wurde nach intraprostatisch geführt und transvesikal von abdominal aus verknotet. Durch leichten Zug an der Naht nach intraprostatisch wurde die Kapsel so nach innen gestülpt. Unter diesem Zug erfolgte die Anlage einer Z‑Naht von extraprostatisch über die Tabaksbeutelnaht. Ein transurethraler 18-Ch-Dauerkatheter (tDK) und ein suprapubischer 12-Ch-Katheter (SPK) wurden eingelegt. Der SPK und die UK wurden transvesikal perkutan ausgeleitet. Die Blase wurde zweireihig verschlossen mit abschließendem Verschluss des Peritoneums.

Nun wurde durch die Kollegen der plastischen Chirurgie ein Gracilisflap aus dem rechten Oberschenkel entnommen und als Interponat zwischen Rektum und Prostatahinterkapsel angelegt und fixiert.

### Postoperativer Verlauf

**Figure 2:**
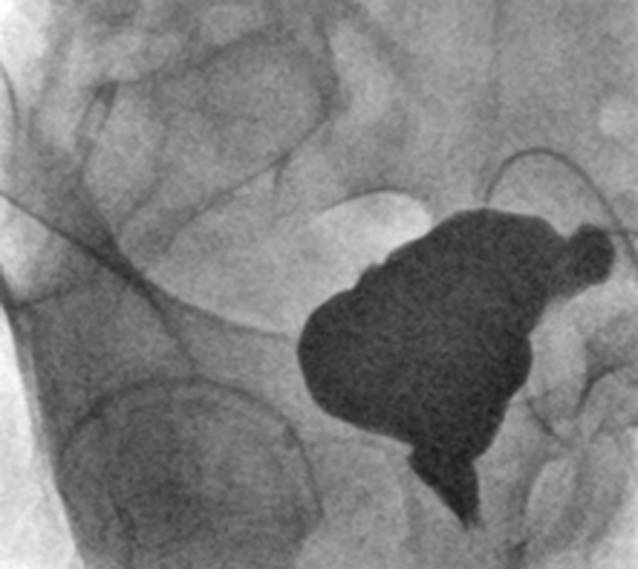

Postoperativ erfolgte eine permanente Niederdruckdauerableitung für insgesamt 3 Wochen. Ein flaues Zystogramm zeigte keine Undichtigkeit, daher wurden die UK entfernt. Jedoch wurde auf eine Füllung mit Druck während des Zystogramms verzichtet und unter Sicherheitsaspekten die Ableitung via SPK und tDK fortgeführt. Im weiteren Verlauf erfolgte am 48. Tag ein erneutes Zystogramm mit Entfernung des tDK (Abb. 2). Nach Miktionsfreigabe mit Miktionskontrolle über den SPK wurde dieser ebenfalls entfernt.

## Ergebnisse

Nach knapp 3 Jahren konnte dem Patienten erstmalig die Harnableitung erfolgreich entfernt werden. In den Miktionstagesprofil Protokollen zeigte sich eine Blasenkapazität bis 600 ml und eine restharnfreier Blasenentleerung mit einer 3‑ bis 4‑maligen Diurie und fehlender Nykturie. Der Patient ist kontinent und hat keinen Vorlagenverbrauch. Aufgrund Compliance war eine standardisierte Erhebung der „Quality of Life“ (QoL) und „International Consultation on Incontinence Questionnaire“ (ICIQ)-Fragebögen nicht möglich.

Im weiteren Verlauf erfolgte die Rückverlagerung des protektiven doppelläufigen Transversostomas.

## Diskussion

Aufgrund von Bestrahlung, Brachytherapie, endoskopischen oder offenen Operation entstehen 60 % der URF iatrogen [2, 3].

Nur in seltenen Fällen werden spontane Remissionen von kleineren Fisteln beobachtet [4]. In der Regel ist eine komplexe operative Therapie notwendig, die einiger Erfahrung bedarf [1].

Ein sog. Standard, welche Operationsmethode bei welcher Fistel anzuwenden ist, besteht nicht. Je nach Fistellokalisation, Operateur und patientenseitiger Begleitumstände müssen unterschiedliche Herangehensweisen in Betracht gezogen werden.

Zu den Grundprinzipien der Fistelchirurgie zählt u. a. die Wahl des Operationszeitpunktes. Ist eine sofortige Revision (bis zum 7. postoperativen Tag) nicht möglich, so muss die ca. 3- bis 6‑monatige Phase der Umbauprozesse abgewartet werden, sodass vitales Gewebe vorliegt [7]. Besonderes Augenmerk liegt auf dem spannungsfreien und mehrschichtigen Verschluss mit Vermeidung übereinander liegender Nähte. Zusätzlich sollte eine konsequente Urindrainage erfolgen und das Gewebe ausreichend, aber nicht übertrieben, mobilisiert werden [8].

Der wahrscheinlich wichtigste Faktor für eine erfolgreiche Fistelsanierung, v. a. bei komplexen Situationen, ist die Verwendung eines Interponats. Ein- oder sogar zweizeitige Techniken mit Trennung des Harntraktes vom Verdauungstrakt im ersten und Verschluss mit Interposition von gutdurchblutetem Gewebe im zweiten Schritt, wurden beschrieben [5].

Bei dem Verschluss einer komplexen URF ist die Verwendung eines vaskularisierten Grafts, wie z. B. dem Gracilisflap, für die Induktion des Heilungsprozesses bedeutend [6]. Auch Omentumflaps (gerade beim abdominalen Zugang) sowie Peritonealflaps haben sich bewährt. Vaskularisierte Grafts wirken außerdem antiinflammatorisch, haben resorbierende Eigenschaften, füllen Hohlräume und verhindern, dass Nahtreihen aufeinander liegen. Erfolgsraten der Fistelverschlüsse unter Verwendung von Interponaten liegen in Reviewstudien zwischen 87,5–93,9 % [11, 12]. Interessanterweise ist der prozentuale Einsatz von Interponaten in High-volume-Zentren (≥ 25 Patienten) am höchsten und liegt bei nahezu 100 % [12].

Liegen urethrorektale Fisteln der prostatischen Harnröhre vor, so wird eine Prostatektomie ggf. mit zusätzlichem Interponateinsatz propagiert. In unserem Falle war dieses Vorgehen ebenfalls geplant, jedoch hätte aufgrund der individuellen Gegebenheiten dies eine Zystektomie zur Folge gehabt.

Das von Naujoks 1955 erstmalig beschriebene Verfahren zur transvaginalen Sanierung einer vesikovaginalen Fistel mit Anlage einer Tabaksbeutelnaht, welche transvesikal und -urethral ausgeleitet wird und für 10 Tage mit einem Dauerzug versehen wird, wurde von Bressel adaptiert und als Operationslehre 1999 veröffentlicht [9, 10].

Dieses Verfahren, in Kombination mit dem etablierten Interponatprinzip, gewährleistete in unserem Fall alle der oben genannten Gegebenheiten, die eine erfolgreiche Fistelsanierung bedingen.

Die Ausbildung einer URF nach einer suprasymphysären Adenomenukleation nach Freyer ist unseres Wissens nach bis dato wissenschaftlich nicht beschrieben und stellt eine wahre Rarität dar. Deutlich häufiger wird eine „Anastomosenrektum“-Fistel nach radikaler Prostatektomie beschrieben.

Auf Basis der Verwendung eines Gracilisflaps als Interponat zum Verschluss der Fistel des hier beschriebenen Falls, konnte durch Adaptation einer etablierten, jedoch nicht weit verbreiteten, chirurgischen Methode des transvaginalen Verschlusses einer vesikovaginalen Fistel nach Bressel-Naujoks, eine potenzielle inkontinente Harn- und Stuhlableitung verhindert werden.

Der hier dargestellte Fall zeigt, dass die Fistelchirurgie, in den Leitplanken festgeschriebener Prinzipien, dem Operateur Flexibilität abverlangt – zum Wohle des Patienten.

## Fazit für die Praxis

- Die Fistelchirurgie verlangt eine gewisse Flexibilität des Operateurs zur Anpassung des operativen Vorgehens je nach intraoperativen Gegebenheiten.
- Die Grundprinzipien der Fistelchirurgie müssen hierbei immer beachtet werden:
- richtiger Operationszeitpunkt,
- spannungsfreier Verschluss der Fistel,
- mehrschichtiger Verschluss der Fistel,
- keine übereinander liegende Nähte, soweit möglich,
- ausreichende, aber nicht übertriebene, Mobilisierung des Gewebes,
- konsequente Urinableitung,
- bei komplexen Fisteln: Verwendung eines Interponats (z. B. Muskelflap, Omentumflap, Peritonealflap).
- Lässt es der individuelle Fall zu, oder macht er es sogar nötig, so sollen auch etablierte Methoden anderer Fistelchirurgien entsprechend adaptiert und verwendet werden.
